# Effectiveness of Leukocyte and Platelet-Rich Fibrin versus Nitrofurazone on Nail Post-Surgery Bleeding and Wound Cicatrization Period Reductions: A Randomized Single Blinded Clinical Trial

**DOI:** 10.3390/jcm8101552

**Published:** 2019-09-27

**Authors:** Xavier Garrido-Castells, Ricardo Becerro-de-Bengoa-Vallejo, César Calvo-Lobo, Marta Elena Losa-Iglesias, Patricia Palomo-López, Emmanuel Navarro-Flores, Daniel López-López

**Affiliations:** 1 Research, Health and Podiatry Unit, Department of Health Sciences, Faculty of Nursing and Podiatry, Universidade da Coruña, 15403 Ferrol, Spain; xavier.garrido.castells@udc.es (X.G.-C.); daniellopez@udc.es (D.L.-L.); 2 Facultad de Enfermería, Fisioterapia y Podología, Universidad Complutense de Madrid, 28040 Madrid, Spain; ribebeva@ucm.es; 3 Faculty of Health Sciences, Universidad Rey Juan Carlos, 28922 Madrid, Spain; marta.losa@urjc.es; 4 University Center of Plasencia, Universidad de Extremadura, 10600 Badajoz, Spain; patibiom@unex.es; 5 Department of Nursing, Faculty of Nursing and Podiatry, Universidad de Valencia, 46100 Valencia, Spain; emmanuel.navarro@uv.es

**Keywords:** inflammation, nail diseases, pain, platelet rich plasma, surgery

## Abstract

**Background:** Leukocyte and platelet-rich fibrin (L-PRF) may be considered a co-adjuvant intervention that may play a key role in blood coagulation and tissue repair after nail surgeries. The aim of this study was to determine the effectiveness of L-PRF versus nitrofurazone on the post-surgical bleeding and wound cicatrization period in patients with bilateral onychocryptosis during surgeries of chemical matrixectomies with 88% phenol solution. **Methods:** A randomized single-blind clinical trial was registered with the European Clinical Trials Database (EudraCT) with identification number 2016-002048-18. Twenty healthy participants with bilateral onychocryptosis (*n* = 40) were recruited and bilaterally received both protocols for both halluces. Patients with a mean age mean of 45.55 ± 12.19 years attended a specialized foot and ankle surgery clinic. Both halluces of each patient were randomized and allocated to receive L-PRF (experimental group; *n* = 20 halluces) or nitrofurazone (control group; *n* = 20 halluces) interventions in conjunction with surgery of chemical matrixectomies with 88% phenol solution for bilateral ingrown of toenail border (medial and lateral). Patients were blinded to their intervention in each hallux. The primary outcome measurement was post-surgical bleeding. The secondary outcome measurements were post-surgical pain intensity, inflammation, infection, analgesic intake, and wound cicatrization period. **Results:** Statistically significant differences (*p* < 0.001) were found between both groups showing a reduction for wound cicatrization period and post-surgical bleeding for the L-PRF intervention with respect to nitrofurazone treatment. The rest of the outcome measurements did not show any statistically significant differences (*p* > 0.05). **Conclusions:** L-PRF rather than nitrofurazone in conjunction with chemical matrixectomies performed with 88% phenol solution reduced the wound cicatrization period and bleeding after nail surgery. Thus, L-PRF may be considered a first-line co-adjuvant intervention for patients who suffer from nail problems, such as onychocryptosis, that require surgical procedures.

## 1. Introduction

Onychocryptosis may be considered a common toenail pathology that seems to be generally presented by adolescents and young adults in the second and third decades of their lives [1]. The most common symptoms of this condition are pain, infection, difficulty in wearing footwear, walking limitations, and a decrease in the quality of life. This condition is considered a therapeutic challenge for surgical specialists, such as dermatologists [2,3,4].

Currently, local application of phenol solution may be considered as the first non-incisional surgical option with success rates of more than 95% with respect to chemical cauterization of onychocryptosis; this option may be considered the gold standard procedure because it shows beneficial effects, such as strong antiseptic properties, production of necrosis during protein coagulation, and reduction of pain due to nerve fiber demyelination in the nail unit [5,6].

Nevertheless, the effects of phenol solution may produce a delay in the period of wound cicatrization (between 21 and 42 days) related to tissue destruction, excessive drainage, and an adverse reaction to the phenol solution, which may produce complications in approximately 15% of applications [7,8,9,10].

Nowadays, leukocyte and platelet-rich fibrin (L-PRF) may be used as a key local treatment in wound healing and tissue repair in patients with various problems [11,12,13].

To our knowledge, there is a lack of studies linking L-PRF to the treatment of chemical matrixectomies that were performed with phenol solution. Despite the general use of nitrofurazone in conjunction with phenol solution [7,8,9,10], L-PRF may be considered a co-adjuvant intervention that may show a key role for blood coagulation and tissue repair after nail surgeries [11,12,13]. Consequently, the aim of this study was to determine the effectiveness of L-PRF versus nitrofurazone on the postsurgical bleeding and wound cicatrization period in patients with bilateral onychocryptosis during surgeries of chemical matrixectomies with 88% phenol solution. We hypothesized that patients who undergo surgery for ingrown toenails with phenol solution in conjunction with L-PRF would show better results with respect to the postsurgical bleeding and wound cicatrization period when compared with nitrofurazone.

## 2. Materials and Methods

### 2.1. Design and Sample

This research consisted of a single-blind clinical trial registered with the European Clinical Trials Database (EudraCT) with identification number 2016-002048-18. In addition, this investigation was conducted in a specialized surgery clinic for the foot and ankle that provides treatments for foot problems in the town of Valencia (Spain). Twenty healthy participants with bilateral onychocryptosis (*n* = 40 halluces) were recruited and bilaterally underwent both protocols for both halluces. Patients with a mean age of 45.55 ± 12.19 years attended a specialized foot and ankle surgery clinic. Both halluces of each patient were randomized by an independent, non-participating clinician and allocated to receive either L-PRF (experimental group; *n* = 20 halluces) or nitrofurazone (control group; *n* = 20 halluces) interventions in conjunction with chemical surgery of matrixectomies with 88% phenol solution for bilateral ingrown toenail border (medial and lateral). Patients were blinded to their intervention in each hallux (right or left), while the surgeon was aware of the intervention applied to each foot. Indeed, the control group consisted of the halluces with onychocryptosis who received surgery with 88% phenol in conjunction with 0.2 g nitrofurazone (Seid S.A, Barcelona, Spain). The experimental group received 88% phenol in conjunction with L-PRF in the contralateral halluces of the same patient with onychocryptosis.

Patient selection was conducted by a random sampling method, and twenty patients (4 male and 16 female) were recruited. The inclusion criteria for all participants consisted of several parameters: (1) Patients diagnosed with bilateral onychocryptosis for both medial and lateral borders of the hallux who were to undergo toenail surgery with phenol; (2) patients without other physical alterations neither medical record disorders; (3) over eighteen years old; and (4) patients who signed the informed consent form. The exclusion criteria consisted of several parameters: (1) Trauma and a history of toenail and foot alterations; (2) known sensitivity to phenol solution; (3) vascular problems; (4) rheumatic conditions and immunocompromised patients; (5) refusal to sign the informed consent form; and (6) limitations with respect to complying with the necessary recommendations for undergoing surgical investigation in ingrown nails.

### 2.2. Leukocyte and Platelet-Rich Fibrin (L-PRF)

L-PRF was administered according to the procedure of Choukroun et al. [14]. This procedure required no anticoagulants or thrombin (or any other gelling agents). A table-top centrifuge (DH Material Médico, Barcelona, Spain) and a collection procedure kit (Vacutainer with citrate concentration of 0.129 mol/L (3.8%), DH Material Médico, Barcelona, Spain) were necessary. The protocol for obtaining L-PRF was very simple. A blood sample without anticoagulants was taken in two 5 mL glass tubes, which were then centrifuged at 3000 rpm (approximately 400× *g*) for 10 min. Absence of the anticoagulant implied quick activation of most of the blood sample platelets in contact with the walls of the glass tubes and release of the coagulation cascade. Fibrinogen was first concentrated in the top part of the tubes before transformation of circulating thrombin into fibrin was obtained. A fibrin clot was then obtained in the middle of the tube (L-PRF) between the red corpuscles at the bottom and acellular plasma at the top.

### 2.3. Procedure

The socio-demographic baseline characteristics (sex, age, weight, height, and body mass index (BMI)) of patients were collected by the same physician who performed the chemical matrixectomy surgery with 88% phenol in a sterile manner according to the protocol of Becerro-de-Bengoa-Vallejo et al. [9]. After the surgery, the hallux was washed with a bristled sterile brush and dual-sided foam saturated with povidone iodine for approximately 5 min.

Next, the toenail was wiped clean with sterile gauze and disinfected with 10% povidone-iodine. Subsequently, 2 mL of 2% mepivacaine (without vasoconstrictor) as a local anesthetic was injected. Next, a digital tourniquet was laid at the base of the hallux of the foot. In addition, the nail plate spicule was retrieved at each border (medial and lateral) with a Kelly hemostat. Residual blood in the zone was cleaned with a sterile gauze and then three rounds were applied with a phenol solution for 1 min at each border of the toenail with the terminal side of the sterile gauze containing a mini osteotome in the border (medial and lateral) in each nail. After this, 15 mL of 70% alcohol was then applied in the nail as a suitable and effective means of diluting and removing any excess or residual phenol from the exposed area by its drag effect [15].

Afterward, L-PRF or nitrofurazone were applied to each border (medial and lateral) of the nail. The digital tourniquet was then removed, and the hallux was wrapped with sterile gauze. All patients received similar analgesic medication prescribed for pain after this surgery. Finally, both treatments were repeated at 48 h and for the next five consecutive days. Afterwards, the treatment consisted of application of povidone iodine until cicatrization of the nail was complete [16].

### 2.4. Outcome Measurements

The primary outcome measurement was post-surgical bleeding, which was categorized as mild (the dressing did not show external spots; only the polypropylene of the dressing was in contact with the wound, and the gauze was in contact with the dressing), moderate (the dressing might have shown slight spots on the back or sides; the non-adherent dressing might have been completely stained and the gauze in contact with it may have been partially stained), or heavy bleeding (the external bandage could be completely or almost completely colored) [17]. The secondary outcome measurements were post-surgical pain intensity as assessed by the visual analogue scale (VAS, showing an intraclass correlation coefficient of 0.97) on the first, second, and third days after surgery [18], post-surgical inflammation as measured by the digital circumference in mm using a flexible ruler (Devon Industries 1-800, Inc., Devon, PA, USA) at the level of the proximal nail fold before and 48 h after surgery during the acute inflammatory phase of healing [16], presence of infection, analgesic intake for post-surgical pain (measured as mg of paracetamol, Termalgin (Novartis Farmacéutica SA, Barcelona, Spain)), and post-surgical wound cicatrization measured as days of cicatrization of the post-surgical wound (days from the surgery to clinical recovery) [16,17].

### 2.5. Sample Size Calculation

The sample size calculation was performed with the ENE 3.0 software (GlaxoSmithKline, Universidad Autónoma de Barcelona, Barcelona, Spain) based on a prior study that investigated the post-surgical hemorrhage difference between partial matrixectomy by non-incisional methods (Suppan I) with post-surgical nitrofurazone or platelet gel treatment [17]. According to these authors, the percentage of bleeding at the final stage of this study was 93.9%. Considering a clinically important reduction of 50% for this percentage, a two-tailed test, an α error of 0.05, and a desired power of 80% (β = 20%), a minimum sample size of 19 halluces per group, experimental and control, was considered.

### 2.6. Ethical and Legal Considerations

The research protocol was approved by the local Ethics and Committee of the San Carlos Clinic Hospital (Madrid, Spain) with identification number of CE 16/401-R and EudraCT record number of 2016-002048-18. All patients who signed the informed consent form were added to this research protocol. Additionally, the guidelines associated with the ethical standards for investigation and experimentation in human participants as reported in the Declaration of Helsinki at the 64th World Medical Assembly (Fortaleza, Brazil) and other international institutional organizations were maintained.

### 2.7. Statistical Analysis

Categorical data were described as frequencies and percentages. The Shapiro Wilk’s test was applied in order to determine the normal distribution of the study data. Quantitative data were described as mean, standard deviation (SD), and 95% confidence interval (CI; lower and upper limits) for parametric data in addition to median and interquartile range (IR) for non-parametric data. Quantitative data were compared by the parametric Student’s *t*-test or non-parametric Mann–Whitney U test for independent samples in order to compare differences between both groups. IBM SPSS Statistic software (v19, SPSS Inc., Chicago, IL, USA) was used for data analyses, and statistically significant differences were set at *p* < 0.05 with a 95% CI.

## 3. Results

### 3.1. Descriptive Data

Both groups were homogeneous due to both halluces (right and left) used for comparison as control and experimental groups. Comparisons between male (*n* = 4) and female (*n* = 16) patients (Table 1) showed statistically significant differences (*p* < 0.05) for height and weight but not for age or BMI (*p* > 0.05).

### 3.2. Outcome Measurements

Statistically significant differences (*p* < 0.001) were shown for a reduction in the post-surgical cicatrization period and a decrease in post-surgical bleeding for the experimental group with respect to the control group (Table 2).

## 4. Discussion

The effectiveness of L-PRF was compared with nitrofurazone for the treatment of chemical matrixectomies with 88% phenol solution. Previous investigations by Córdoba-Fernández et al. reported that application of platelet-rich plasma in onychocryptosis contributed to the improvement in acute inflammation, but a reduction in postoperative pain was not achieved [16,17].

The findings of our novel study may be considered as the first results showing that the use of L-PRF treatment for the chemical matrixectomies with 88% phenol solution improved the cicatrization period and also reduced nail bleeding during the post-surgical period compared with nitrofurazone. In addition, all patients showed higher satisfaction with L-PRF treatment for their ingrown toenails suggesting that L-PRF may be used as a first-line treatment in these patients.

Besides, these findings were in line with finding from a study by Munoz et al. in which an improvement in the periodontal post-surgical period showing decreased pain, minor inflammation, and infection with the use of L-PRF in these patients was demonstrated [19].

Nevertheless, our research presented some limitations that should be acknowledged. First, new investigations should compare L-PRF and phenol solution in a double-blind controlled clinical trial using a placebo intervention. A larger and more diverse sample size (patients from other countries) would improve the strength of this investigation. Second, despite 70% alcohol was applied [15], it is not a phenol neutralizer and could be removed from this procedure and replaced by sterile saline solution [20]. Finally, this highlights the need for further research with the use of L-PRF to treat nail surgery in addition to foot and ankle problems.

## 5. Conclusions

L-PRF versus nitrofurazone interventions in conjunction with chemical matrixectomies with 88% phenol solution was shown to reduce the wound cicatrization period and bleeding after nail surgery. Thus, L-PRF may be considered as a first-line co-adjuvant intervention for patients who suffer from nail problems, such as onychocryptosis that require surgical intervention.

## Figures and Tables

**Table 1:** Socio-demographic characteristics of the study sample by sex distribution.

Characteristics	Male (*n* = 4) Mean ± SD (95% CI)	Female (*n* = 16) Mean ± SD (95% CI)	Total (*n* = 20) Mean ± SD (95% CI)	*p*-Value *
Age (years)	48.63 ± 14.68 (38.45–58.79)	44.78 ± 11.63 (40.75–48.81)	45.55 ± 12.19 (41.77–49.33)	0.432 *
Weight (kg)	72.88 ± 8.25 (67.16–78.59)	63.66 ± 7.81 (60.95–66.36)	65.50 ± 8.64 (62.82–68.18)	0.005 *
Height (m)	1.80 ± 0.06 (1.76–1.85)	1.63 ± 0.06 (1.60–1.66)	1.66 ± 0.10 (1.63–1.69)	0.001 **
BMI (kg/cm^2^)	22.41 ± 2.18 (20.91–23.92)	23.89 ± 3.99 (22.50–25.27)	23.59 ± 3.72 (22.44–24.74)	0.324 *

Abbreviations: BMI, body mass index; CI, confidence interval; SD, standard deviation. * Mann–Whitney U test, ** Student *t* test for independent sample. Statistically significant differences were set at *p* < 0.05 with a 95% CI.

**Table 2:** Comparisons for outcome measurements between both treatment groups.

Outcome Measurements	Control Group (*n* = 20)	Experimental Group (*n* = 20)	*p*-Value
	Mean ± SD (95% CI) Median (IR)	Mean ± SD (95% CI) Median (IR)	
Cicatrization period of the post-surgical wound (days)	22.10 ± 2.69 (20.92–23.28) 21 (2)	14.53 ± 1.66 (13.62–15.08) 14 (2)	<0.001 *
Post-surgical bleeding (Mild = 0; moderate = 1; heavy = 2)	1.25 ± 0.55 (1.01–1.49) 1.00 (1)	0.15 ± 0.37 (−0.01–0.31) 0.00 (0)	<0.001 *
Post-surgical inflammation (mm; digital circumference)	85.00 ± 3.81 (83.33–86.67) 84.50 (3.5)	84.20 ± 5.54 (81.77–86.62) 84.50 (3.25)	0.925 *
Post-surgical pain at 1st day (VAS)	3.30 ± 2.66 (2.14–4.46) 3.00 (4.25)	3.30 ± 2.54 (2.19–4.41) 3.00 (2.5)	1.000 *
Post-surgical pain at 2nd day (VAS)	1.65 ± 1.84 (0.84–2.46) 1.00 (3.25)	1.45 ± 1.82 (0.65–2.25) 0.50 (2.25)	0.758 *
Post-surgical pain at 3rd day (VAS)	0.60 ± 1.23 (0.06–1.14) 0.00 (1)	1.05 ± 1.90 (0.22–1.88) 0.00 (1.25)	0.678 *
Post-surgical analgesic intake (mg)	725 ± 1057.24 (261.65–1188.34) 0 (1000)	325 ± 591.05 (65.96–584.03) 0 (500)	0.147 *

Abbreviations: CI, confidence interval; IR, interquartile range; SD, standard deviation; VAS, visual analogue scale. * Mann–Whitney U test. Statistically significant differences were set at *p* < 0.05 with a 95% CI.
